# Advances in Diagnostic and Interventional Catheterization in Adults with Fontan Circulation

**DOI:** 10.3390/jcm13164633

**Published:** 2024-08-07

**Authors:** Yassin Belahnech, Gerard Martí Aguasca, Laura Dos Subirà

**Affiliations:** 1 Adult Congenital Heart Disease Unit, Cardiology Department, Vall d’Hebron University Hospital, Vall d’Hebron Research Institute, Universitat Autònoma de Barcelona, 08035 Barcelona, Spain; yassin.belahnech@gmail.com (Y.B.); drgmarti@googlemail.com (G.M.A.); 2 Centro de Investigación Biomédica en Red en Enfermedades Cardiovasculares (CIBERCV), Instituto de Salud Carlos III, 28029 Madrid, Spain; 3 European Reference Network for Rare, Low-Prevalence, or Complex Diseases of the Heart (ERN GUARD-Heart), Coordinating Center in Amsterdam, 1105 AZ Amsterdam, The Netherlands

**Keywords:** congenital heart disease, single ventricle, Fontan circulation, catheterization, hemodynamic assessment, percutaneous interventions

## Abstract

Over the past five decades, the Fontan procedure has been developed to improve the life expectancy of patients with congenital heart defects characterized by a functionally single ventricle. The Fontan circulation aims at redirecting systemic venous return to the pulmonary circulation in the absence of an impelling subpulmonary ventricle, which makes this physiology quite fragile and leads to several long-term complications. Despite the importance of hemodynamic assessment through cardiac catheterization in the management and follow-up of these patients, a thorough understanding of the ultimate functioning of this type of circulation is lacking, and the interpretation of the hemodynamic data is often complex. In recent years, new tools such as combined catheterization with cardiopulmonary exercise testing have been incorporated to improve the understanding of the hemodynamic profile of these patients. Furthermore, extensive percutaneous treatment options have been developed, addressing issues ranging from obstructive problems in Fontan pathway and acquired shunts through compensatory collaterals to the percutaneous treatment of lymphatic circulation disorders and transcatheter edge-to-edge repair of atrioventricular valves. The aim of this review is to detail the various tools used in cardiac catheterization for patients with Fontan circulation, analyze different percutaneous treatment strategies, and discuss the latest advancements in this field.

## 1. Introduction

Fontan circulation is a palliative surgical technique developed to improve the life expectancy of children born with complex congenital heart diseases that share a functionally single ventricle as a common feature. This technique involves redirecting systemic venous return to the pulmonary arteries without a propelling subpulmonary ventricle. Since its inception in 1971 [1], the technique has undergone numerous modifications, evolving from atriopulmonary connections to cavopulmonary connections, at present through extracardiac conduits [2].

**Figure 1:**
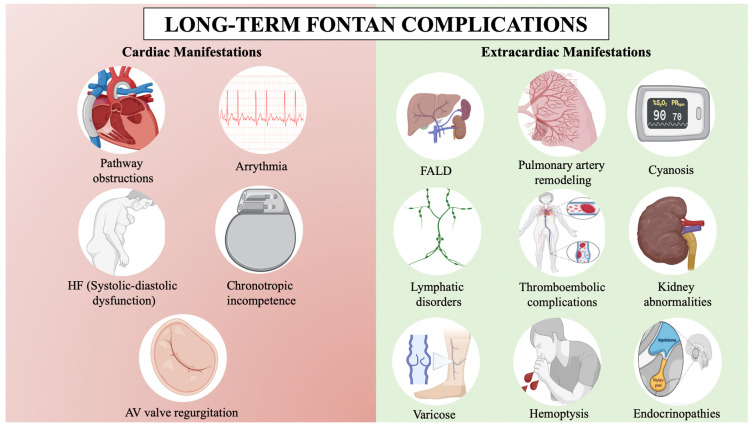
Long-term Fontan complications. AV: Atrioventricular, FALD: Fontan-associated liver disease, HF: Heart failure. BioRender.com has been used for the creation of this figure (Agreement number: RI275JMOWR).

Fontan circulation improves quality of life, exercise capacity, and life expectancy, with a 30-year survival rate exceeding 85%, but it is not without serious complications [3,4] (Figure 1). Complications are due to the passive and non-pulsatile nature of the pulmonary blood flow, which leads to a progressive increase in central venous pressure to propel blood through the lungs, causing systemic venous congestion. Additionally, it is hypothesized that these patients have a higher incidence of pulmonary vascular disease due to endothelial dysfunction and nitric oxide pathway dysregulation in the absence of a pulsatile flow [5]. The absence of a subpulmonary ventricle makes the central venous pressure highly sensitive to small changes, whether due to obstructions in the circuit, slight increases in pulmonary vascular resistance (PVR), or in single ventricle filling pressure [5,6]. As a result, arrhythmias, heart failure, Fontan-associated liver disease (FALD), lymphatic disorders, or thromboembolic phenomena may ensue [7]. Fontan failure leads to increased mortality and only heart transplantation or, in selected cases, Fontan takedown may be an option [8].

Cardiac catheterization, both from a diagnostic and therapeutic standpoint, is a cornerstone in the monitoring and treatment of Fontan patients, and up to 50% of adult patients require some sort of percutaneous intervention [9]. Cardiac catheterization is recommended for symptomatic patients with primary symptoms such as unexplained edema, exercise deterioration, new-onset arrhythmia, cyanosis, hemoptysis, and protein-losing enteropathy. However, in asymptomatic patients, cardiac catheterization remains a controversial topic due to the lack of clear evidence of its benefit and the absence of a uniform recommendation among clinical guidelines [2,10].

This review aims to provide a comprehensive overview of both diagnostic and therapeutic usefulness of cardiac catheterization in patients with Fontan circulation, and it aims to update the most relevant advances in this field.

## 2. Diagnostic Procedures

Table 1 summarizes the main diagnostic tools.

### 2.1. Hemodynamic Assessment at Rest

Hemodynamic studies in patients with Fontan circulation aim to provide a comprehensive assessment of the Fontan venous circuit, pulmonary vasculature, and ventricular function. This entails a thorough examination of venous, pulmonary, and ventricular filling pressures, along with an estimation of cardiac output (CO) and PVR [5].

#### 2.1.1. Invasive Pressure Assessment

Central venous pressure in healthy individuals ranges from 2–6 mmHg [11]. However, in non-failing Fontan patients, it is markedly higher (8–12 mmHg) [12], and differences may be more pronounced during exertion [13]. Due to the lack of a subpulmonary ventricle, in the absence of a circuit stenosis, central venous pressure and pulmonary arterial pressure are equal and lack pulsatility. Venous pressure in Fontan patients is crucial for ensuring adequate ventricular preload and, consequently, proper CO [5]. However, elevated venous pressure values exceeding 15 mmHg at rest in Fontan patients correlate with a poorer prognosis and increased mortality rates [14]. Venous pressure must be recorded across all venous and pulmonary circuits to rule out possible stenosis, and should include the superior and inferior vena cava, both pulmonary branches, and the Fontan conduit, where resting gradients of 2 mmHg or more may indicate a stenosis significant enough to impair the proper functioning of the Fontan circulation [15,16]. Prior to surgical correction, univentricular hearts are usually exposed to volume overload due to previous palliative surgeries. The reduction in ventricular preload caused by superior cavopulmonary connection (bidirectional Glenn) and Fontan surgeries may lead to a reduction in CO and to an increase in peripheral vascular resistance, leading to diastolic dysfunction, thus compromising the passive forward flow of Fontan circuit. During catheterization, ventricular filling pressures, which should remain below 10–12 mmHg [14,16], may be recorded using pulmonary artery wedge pressure (PAWP), systemic atrial pressure if accessible through a previous fenestration, or, in the absence of atrioventricular valvulopathies, using the end diastolic ventricular pressures.

Despite the difficulty in establishing normal values, a study of long-term excellent Fontan survivors, who were free of complications and had good ejection fraction, reported the following after undergoing periodic cardiac catheterizations over 15 years: an end-diastolic pressure of 6.8 ± 1.6 mm Hg, a central venous pressure of 10.1 ± 1.8 mm Hg, and a cardiac index (CI) of 2.6 ± 0.6 L/min/m^2^ [12].

The transpulmonary gradient (TPG) is the difference between mean pulmonary artery pressure (equivalent to central venous pressure in Fontan patients without pathway obstructions) and ventricular filling pressure. An elevated TPG value may indicate pulmonary vascular remodeling [11]. An advantage of TPG is that it is not influenced by the estimation of CO. However, reference values for TPG are not clearly defined for patients with Fontan circulation. Some authors propose a TPG ≥ 7 as a value that could warn of a pre-capillary pulmonary vascular disease [17,18].

In patients with aortic arch reconstructions, such as those who have undergone Norwood or Damus-Kaye-Stansel procedures, it is crucial to conduct a pullback assessment of the gradient across the reconstructed aortic arch. Gradients exceeding 10–15 mmHg should be verified through simultaneous recording, prompting consideration for treatment of a potential arch obstruction [19].

FALD represents a spectrum of liver disorders ranging from chronic fibrosis to advanced cirrhosis [20]. Portal hypertension can be assessed hemodynamically through catheterization by determining the hepatic venous pressure gradient (HVPG), which is obtained from the difference between the hepatic vein wedge pressure and the free hepatic venous pressure [21]. Elevated HVPG may indicate portal hypertension. Although HVPG > 12 mmHg has been correlated with the presence of cirrhosis and higher mortality in non-Fontan patients [22,23], its clinical usefulness in Fontan patients has not been demonstrated, as it does not allow to identify patients with extensive FALD, and it should not be used as a screening method prior to a liver biopsy [24].

#### 2.1.2. Cardiac Output Flow Assessments

It is essential to ascertain CO in patients with Fontan circulation, but its measurement is subject to limitations [25]. Typically, these patients tend to have lower resting CO and a blunted increase with exercise, aggravated in those with chronotropic incompetence [26]. There is no clear consensus on reference values for CI in Fontan patients, but many authors suggest that it does not usually exceed 2.5–3 L/min/m^2^ [12,27]. Interestingly, higher CO at baseline, when observed along with high venous pressures, has been associated with worse prognosis and higher mortality [28]. A plausible explanation is that long-term venous congestion leads to advanced stages of FALD, which results in a reduction in systemic vascular resistance and a hyperdynamic state [29].

Thermodilution is not a valid method for calculating CO in Fontan circulation patients due to the absence of a subpulmonary ventricle. Instead, estimation based on the Fick principle is utilized [30], with the following simplified formula (without considering dissolved oxygen):

CO = VO_2_/([O_2_]_a_ − [O_2_]_v_)

where CO represents cardiac output; VO_2_ is oxygen consumption; [O_2_]_a_ is arterial oxygen concentration; and [O_2_]_v_ is venous oxygen concentration.

The main limitation is the direct measurement of VO_2_, which requires gas consumption measurement equipment, is generally not available in routine clinical practice [31]. Therefore, indirect calculation via the Fick method is often used, employing pre-established tables such as those by Lafarge [32], although these have not been specifically validated in Fontan circulation, and may not be valid due to differences in body composition and sarcopenia [33].

Challenges also arise in calculating oxygen concentrations ([O_2_]) [34], summarized in the following as:

[O_2_] = 1.36 (mlO_2_/gHb) × [Hb] (g/L) × Sat (%)

where 1.36 stands as a constant representing the hemoglobin capacity to transport oxygen in the blood; [Hb] is the concentration of hemoglobin; and Sat represents the oxygen saturation in hemoglobin. Oximetry is used to determine oxygen saturation, which can vary within the same individual at different times, in addition to limitations associated with estimating mixed venous saturation. Generally, in Fontan patients, there is preferential flow from the inferior vena cava to the left lung and from the superior vena cava to the right. Additionally, many patients have aortopulmonary collaterals that can cause an increase in oxygen saturation in the pulmonary artery. For this reason, mixed venous saturation (MV_sat_) should be based on the saturation of both caval veins, either by averaging them or, more accurately, by applying the Flamm equation [35]:

MV_sat_= [(3 × SVC_sat_) + IVC_sat_]/4

where SVC_sat_ represents the oxygen saturation of the superior vena cava and IVC_sat_ represents the oxygen saturation of the inferior vena cava.

In addition to the inherent limitations in calculating CO, specific features of Fontan circulation complicate interpretation further [36]. Coronary venous return is not redirected to pulmonary circulation, so some degree of systemic desaturation is common and may add inaccuracy in oxygen saturation measurements. Additionally, some patients may have fenestrations or venovenous collaterals, which create a right-to-left shunt that increases CO at the expense of cyanosis, thus adding complexity and inaccuracy to calculations of systemic and pulmonary output. Major aortopulmonary collateral arteries (MAPCAs), particularly when large and non-restrictive, can influence segmental flows and result in varying TPG, hindering the calculation of segmental vascular resistances via catheterization. Other challenges arise from measurements taken under sedation or general anesthesia, particularly in pediatric patients, and overnight fasting as well may both alter their hemodynamic state, making the interpretation of results less reliable [36]. In addition, in patients receiving supplemental oxygen with a FiO_2_ > 30%, dissolved oxygen in the blood should be considered in the calculation of arterial and venous concentrations.

Combining cardiac catheterization with cardiac resonance imaging could be a partial solution to mitigate certain errors in CO calculation, especially in patients with segmental flows due to MAPCAs [37,38]. However, the logistical feasibility of performing both procedures simultaneously is very limited.

#### 2.1.3. Pulmonary Vascular Resistance

By dividing the TPG by the CO or the TPG by the CI, PVR and indexed pulmonary vascular resistance (PVRI) are obtained, respectively [11]. Consequently, assumptions about CO can significantly contaminate the calculations of PVR. Pulmonary vascular remodeling in Fontan patients has been linked to lack of pulsatility, reduced capillary recruitment and greater endothelial dysfunction [39], which may cause structural changes in the vascular bed, as evidenced by extensive vascular fibrosis in autopsies from deceased patients with Fontan failure [40].

PVR may limit ventricular preload despite increasing central venous pressure, thus decreasing CO. It has been observed that PVRI > 2 WU*m^2^ along with a CI < 2.5 L/min/m^2^ are independent risk factors for Fontan failure at 5 years [41]. These values may apply to patients with a body surface area (BSA) near normal. However, for individuals with extremes of BSA, no data currently exist for the Fontan circulation, but by consensus, a cut-off for PVR > 2.3 WU is suggested [16]. It is assumed that pulmonary vascular remodeling appears in the long term after Fontan palliation, as these patients display higher PVR after heart transplantation compared to recipients transplanted shortly after Fontan completion [42].

### 2.2. Fluid Challenge, Passive Leg-Lifting and Exercise Test

As observed in patients with heart failure, filling pressures in Fontan circulation may be normal at rest but can increase markedly if a provocative testing is performed with saline load, a passive leg raise or exercise [43,44].

The fluid overload test consists of the administration of 500 mL or 7 mL/kg of saline load within 5–10 min aiming to unmask diastolic dysfunction [45]. As contrast is administered for angiographic studies, some centers complete with saline the remaining weight-based volume until 500 mL are reached. In heart failure studies, a PAWP above 18 mmHg after saline load has been established as diagnostic for ventricular diastolic dysfunction [46]. A similar volume load can be achieved with a passive leg raise to 45 degrees for 1–2 min, and a similar cutoff is used [47]. However, although these maneuvers can provide relevant information for the diagnosis of diastolic dysfunction in Fontan patients [48], these cut-off points have not yet been validated in the Fontan population.

Exercise can be performed in the catheterization laboratory, alone or in combination with a cardiopulmonary exercise test (CPET), to stress pulmonary vasculature. Although no clearly established cutoff points exist, Table 2 provides an approximation of normal values at rest and after supine cycling exercise, based on results from various studies. A freewheel stage is recommended, followed by an incremental load with an increase per stage of 10–25 W every 1–2 min, aiming to reach maximum effort in 8–12 min [49]. This method not only allows for the determination of Fontan central venous pressure during exertion, but it also enables the estimation of V02 and, therefore, the measurement of CO using direct Fick method. A slope > 3 mmHg/L/min during exercise between the increase in mean pulmonary artery pressure over pulmonary flow (ΔmPAP/ΔpCO) defines the development of exercise-induced pulmonary hypertension [11], and correlated with peak VO_2_, N-terminal pro b-type natriuretic peptide and liver stiffness better than PVRI > 2 WU*m^2^ at rest [43]. Conversely, a slope of > 2 mmHg/L/min between the increase in PAWP over systemic flow (ΔPAWP/ΔsCO) identifies patients with diastolic dysfunction [11]. Despite limitations in measuring systemic and pulmonary flow, particularly in the presence of shunts, these cut-offs also retain their usefulness in Fontan patients [43,50]. In heart failure patients, an increase in PAWP > 25 mmHg during exercise is also used for diagnosing diastolic dysfunction [51,52], but a lower cut-off of 20 mmHg with mPAP > 25 mmHg may apply to Fontan patients [44].

**Figure 2:**
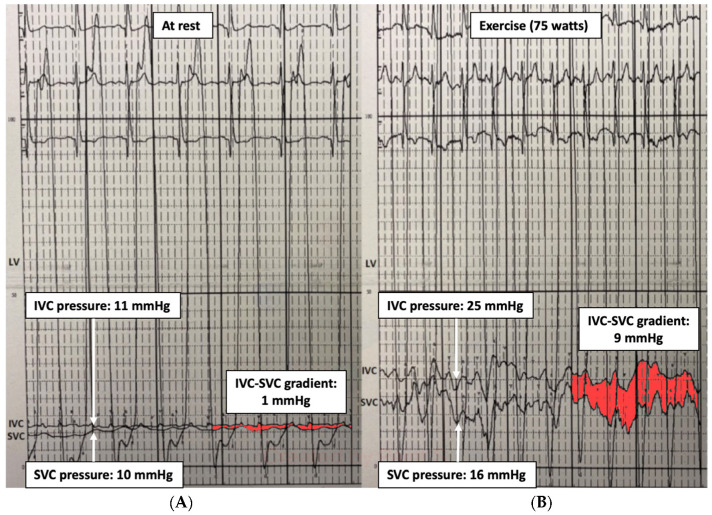
Fontan gradient between the superior and inferior vena cava unmasking an obstructive gradient in the Fontan conduit during exercise. (**A**) SVC-IVC gradient at rest of 1 mmHg. (**B**) SVC-IVC gradient during exercise of 9 mmHg. LV: single ventricle, SVC: superior vena cava, IVC: inferior vena cava.

Therefore, an invasive cardiopulmonary exercise test may identify the hemodynamic pattern responsible for the limited exercise capacity: (1) hidden diastolic dysfunction, (2) pulmonary vascular remodeling, (3) chronotropic incompetence, and (4) inability to increase CO. It can also be useful for detecting conduit stenosis unnoticed at rest by revealing a pressure gradient between the inferior vena cava and superior vena cava (IVC-SVC gradient) (Figure 2), especially if a small gradient (1–2 mmHg) has been observed at baseline. It has been observed that an exercise IVC-SVC gradient correlates well with the minimum Fontan diameter and conduit size, and a Fontan circuit ≥ 16 mm do not exhibit an IVC-SVC gradient > 5 mmHg during exercise [15].

### 2.3. Angiography Studies

**Figure 3:**
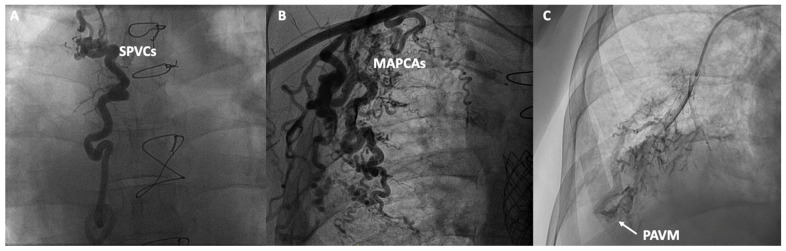
Contrast angiographic studies: (**A**) Systemic to pulmonary venous collaterals (SPVCs); (**B**) Major aortopulmonary collateral arteries (MAPCAs); (**C**) Macrovascular pulmonary arteriovenous malformation (PAVM).

In addition to functional evaluation, catheterization allows for anatomical assessment through contrast angiography studies aimed at evaluating various structures and anomalies associated with Fontan. In the venous territory, angiographies of the inferior and superior vena cava can be conducted, along with selective angiographies of the pulmonary arteries or the azygos system, aiming to detect obstructions, stenoses, the presence of unknown fenestrations, systemic-to-pulmonary venous collaterals (SPVCs), or pulmonary arteriovenous malformations (PAVM). In the arterial territory, aortography can be performed to assess the presence of aortic insufficiency, coarctations, or MAPCAs. In specific cases, ventriculography can evaluate the presence of atrioventricular valve insufficiency or the systolic function of the single ventricle (Figure 3). However, non-invasive imaging using cardiac magnetic resonance, ultrasound and cardiac CT have displaced classic angiographic studies [53].

### 2.4. Intrapulmonary Shunts

The assessment of intrapulmonary shunts via angiography is limited, with the exception of macrovascular PAVM (Figure 3). To identify diffuse and microvascular PAVM, which are much more common, agitated saline is selectively administered into each pulmonary artery to prevent contamination due to fenestrations or SPVCs, and echocardiography is observed to assess the presence of bubbles in the ventricle. Severity is graded as non-mild, moderate, or severe, with the latter two indicating PAVM [54].

## 3. Advances in Interventional Catheterization

Table 3 summarizes interventional therapeutic options for patients with Fontan Circulation.

### 3.1. Management of Fontan Pathway Obstructions

**Figure 4:**
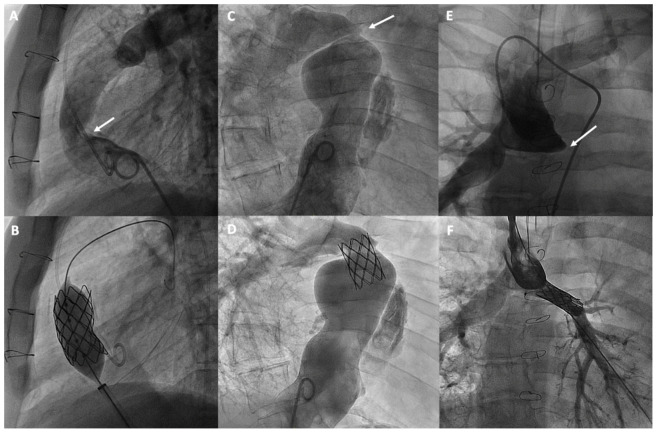
Percutaneous treatment of Fontan pathway obstructions with stent angioplasty on (**A**,**B**) an obstructed extracardiac Fontan conduit, on (**C**,**D**) an atriopulmonary anastomosis in classic Fontan, and on (**E**,**F**) a complete occlusion of the left pulmonary branch artery. The arrows in each figure indicate the point of obstruction or occlusion.

Obstructive issues in Fontan circulation can develop at various levels. Based on anatomy and the type of surgery, it is crucial to distinguish between the different types of obstructions that can occur in the atriopulmonary connections or cavopulmonary circuits, whether in the lateral tunnel, the extracardiac conduit, or their anastomoses. Additionally, these obstructions can occur in the bidirectional Glenn anastomosis, as well as in the pulmonary arteries (Figure 4). A typical site of obstruction is related to previous Blalock-Taussig shunts. Several mechanisms have been identified as causes of these obstructions in Fontan conduit, including a progressive reduction in diameter due to intimal thickening, mismatches or kinking due to physical growth, phenomena such as thrombosis, surgical scarring, or extrinsic compression [55]. However, obstructions that develop in the pulmonary vascular bed may be attributable to surgical factors or to insufficient growth of the pulmonary arteries, which can be favored by the non-pulsatile circulation characteristic of the Fontan procedure [56,57].

Angiographic studies or non-invasive imaging tests, such as cardiac magnetic resonance (MR) or computed tomography, can facilitate the diagnosis, localization, and assessment of the severity of the obstruction to plan their treatment [58]. There are uncertainties regarding which obstructions should be treated and how to assess their severity. Small gradients may be functionally relevant, and if they are accompanied by significant stenosis, they should be considered for treatment. Functional assessment through exercise catheterization can reveal gradients that are otherwise insignificant at baseline [15].

Currently, stenting is the most commonly used technique, with a high success rate and safety, for treating obstructions in the various substrates of the Fontan [59] and proximal pulmonary branches, leaving balloon angioplasty for selected cases or for treating more distal pulmonary arteries [60] (Figure 4). Pre-dilation is generally not recommended, especially if there is a risk of rupture. However, high-pressure post-dilation is advised to optimize stent placement in cases of under-expansion. The use of covered stents is recommended for calcified conduits at risk of rupture, while bare-metal stents are preferred for treating pulmonary branches to avoid the risk of jailing side branches [60].

### 3.2. Management of Fenestrations

Fenestration in the Fontan procedure is primarily a surgical technique, though it can also be achieved percutaneously, creating a controlled bypass between the systemic venous return and the common atrium. This deliberate opening is essential for mitigating potential severe congestion or reduced CO, particularly in the critical early post-operative phase [5]. However, one of the long-term drawbacks of fenestration can be exercise-induced desaturation or, in more severe cases, symptoms of cyanosis. Additionally, there is also a risk of paradoxical embolism [61]. Therefore, there is the option to create or close a fenestration depending on the need of the patient.

In patients with high venous pressures where the creation of a percutaneous late fenestration may be indicated, the procedure can be performed by puncturing using a Brockenbrough needle or radiofrequency [62,63]. The anatomical location of the puncture will vary, depending on the surgical substrate, with the goal of connecting to the common atrium. Typically, the placement of a stent or a fenestrated occluder ensures that the fenestration remains open [64,65].

**Figure 5:**
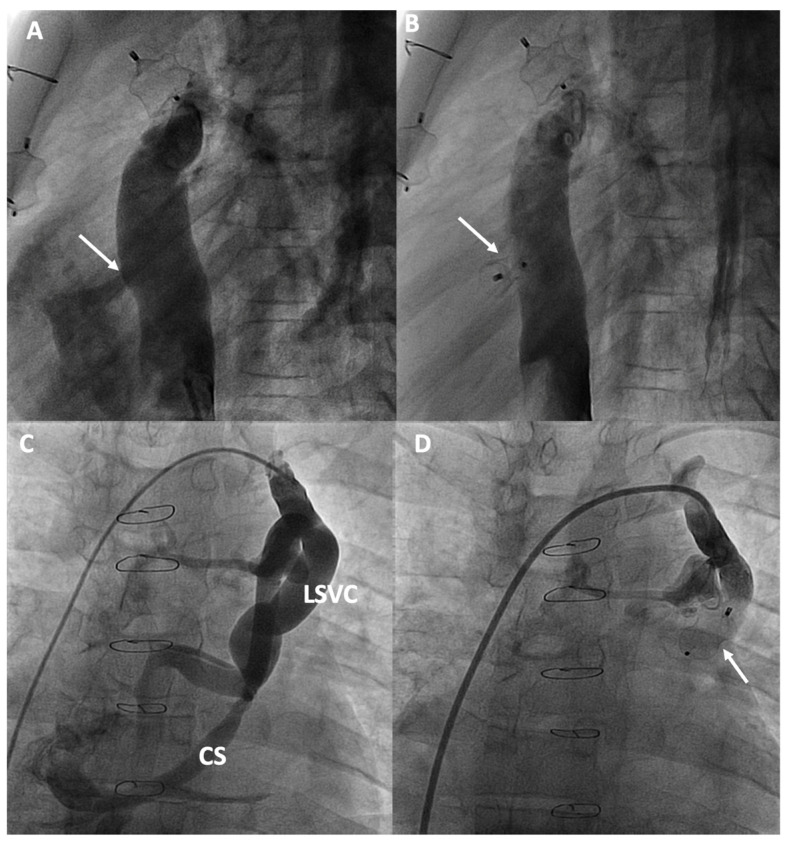
Percutaneous intervention in cyanotic patients due to right-to-left shunt: (**A**,**B**) Fenestration closure; (**C**,**D**) Veno-venous shunt closure from a persistent left superior vena cava (LSVC) to the coronary sinus (CS). The arrows in each figure indicate the shunt and its closure with an occlusion device.

The use of different transcatheter occlusion devices for fenestration closure, such as the Amplatzer Septal Occluder (ASO), Amplatzer Vascular Plug II (AVP II) or the Amplatzer duct Occluder II (ADO II), has been successfully employed, yielding excellent follow-up results [66,67,68] (Figure 5A,B). In instances involving a fenestrated conduit, the feasibility of implanting a covered stent to exclude the fenestration may be assessed, particularly beneficial if conduit stenosis is present [69]. One of the key considerations when deciding whether to close a fenestration in cyanotic patients is the potential risk of hemodynamic deterioration. Therefore, in high-risk cases, the evaluation of filling pressures during temporary balloon occlusion of the fenestration may be helpful [70]. If hemodynamic deterioration occurs following fenestration closure, percutaneous removal of the occlusive device could be considered.

### 3.3. Management of Collaterals

Patients with Fontan circulation often present MAPCAs and/or SPVCs [71,72]. However, there are controversies regarding their clinical and hemodynamic impact. Additionally, there are often difficulties in quantifying the blood flow dependent on these collaterals angiographically, and some authors advocate for a precise study using angio-MR [37,38]. All of these factors complicate and limit the establishment of defined indications for their embolization.

MAPCAs are connections between branches of the aorta and the pulmonary vascular bed, commonly found in Fontan patients, believed to form due to factors like cyanosis or reduced pulmonary flow volume. Despite MAPCAs developing to improve oxygenation in poorly perfused areas of the lung, they can lead to a higher risk of hemoptysis and volume overload on the functionally single ventricle, which can increase the risk of heart failure [73]. SPVCs are connections that typically form between the veins of the cava system or the azygos-hemiazygos system and the pulmonary veins/common atrium, creating a right-to-left shunt. Patients with persistent left superior vena cava can also produce a shunt similar to that of SPVCs through the coronary sinus (Figure 5C,D). More than half of the patients undergoing catheterization may present with SPVCs [72]. Although it is believed that these collaterals form as a result of increased central venous pressure and serve to decompress the venous system, if the shunt flow is significant, it can result in symptoms of cyanosis [74].

The percutaneous treatment for both MAPCAs and SPVCs typically involves embolization. To prevent recanalization, collateral vessels must be occluded from distal to proximal. There are different embolization techniques such as the use of various types of coils and microcoils, microspheres like polyvinyl alcohol (PVA), and liquid embolic systems like Onyx™. Additionally, a variety of vascular plug devices can also be employed [75].

### 3.4. Management of Lymphatic Circulation

The complex lymphatic anatomy, in general terms, consists of the thoracic duct, which begins with a fusiform dilation, known as the cisterna chyli, and drains into the venous circulation at the junction between the left subclavian and left internal jugular veins. It collects most of the lymph, except from the upper right part of the body, which is collected by the right lymphatic duct. Additionally, there are multiple lympho-venous connections in the thorax and abdomen, and a large portion of the total lymph is produced by the liver and intestinal lymphatics [76].

It is believed that the increase in systemic venous pressure has a dual effect, impeding normal lymphatic drainage and also leading to significant increases in lymph production, particularly at the liver [77]. Over time, an imbalance in lymph production and drainage can overload the lymphatic system, leading to its decompression into extralymphatic compartments, potentially causing various disorders such as protein-losing enteropathy, plastic bronchitis, edema, ascites, or chylothorax, which can increase both morbidity and mortality [78].

Historically, the lymphatic system has been challenging to study and assess, leading to the frequent underdiagnosis of related abnormalities. Recent advances in imaging techniques have significantly improved our ability to visualize and characterize the central lymphatic system in detail. Notably, non-contrast MR lymphangiography and intranodal dynamic contrast MR lymphangiography (IN-DCMRL) have become central to these advancements. The former employs T2 sequences to image slow-moving fluids but lacks dynamic data, which can limit its utility [79]. The latter technique involves inserting needles into lymph nodes or ducts, using iodinated contrast or ultrasound for accurate positioning, and provides comprehensive visualization of lymphatic flow and organ perfusion [80]. In addition to IN-DCMRL, intrahepatic dynamic contrast MR lymphangiography (IH-DCMRL) has also been described, allowing for the targeting of hard-to-reach areas of the lymphatic system [81,82,83]. Finally, the use of contrast-enhanced ultrasound has also been recently described for assessing thoracic duct patency and may be a useful tool for interventional procedures [84].

In highly selected patients, interventional treatment may be an option and can be summarized in techniques intended for embolization of abnormal lymphatic vessels and those aimed at decompressing the lymphatic system. Periportal selective lymphatic embolization and percutaneous thoracic duct lymphatic fistulas embolization have proven to be useful for improving protein-losing enteropathy and plastic bronchitis respectively [85,86]. Access to an inguinal or periportal lymph node is achieved under ultrasound guidance, followed by a lymphangiogram with contrast to visualize the central lymphatic system. The target lymphatic vessel is accessed via transabdominal puncture, and various embolization techniques are employed based on the anatomy being addressed, including lymphatic embolization with lipiodol, coils, glue, and sometimes even placement of a covered stent [78]. Percutaneous decompression of the thoracic duct has been recently described [87]. The objective is to promote drainage of the thoracic duct by creating a shunt and decompressing it into a lower pressure chamber. In cases where the thoracic duct drains normally into the left venous angle, the technique involves creating a connection through a puncture between the left superior vena cava and the left atrium, either directly or through the coronary sinus, and occluding the innominate vein [87]. While symptom improvement has been observed with these techniques, the long-term outcomes remain unknown.

### 3.5. Other Interventional Techniques

#### 3.5.1. Transcatheter Edge-to-Edge Atrioventricular Valve Repair

Atrioventricular valve failure is notably prevalent in patients who have undergone single ventricle palliation, particularly those with a common atrioventricular valve [88]. In severe cases, this condition significantly increases the risk of Fontan circuit failure. Traditionally, surgical interventions, including valve repair or replacement, have been the primary treatments [88]. However, the advent of transcatheter edge-to-edge repair (TEER) offers a less invasive alternative, potentially reducing the associated surgical risks, which could be particularly advantageous for patients with Fontan circulation. One of the challenges of TEER is the anatomical variability, as the atrioventricular valve may be common, tricuspid, or mitral. Favorable short-term data have been reported in patients with systemic tricuspid regurgitation [89]. Another significant consideration is that accessing the systemic atrium can be challenging and might require puncturing the Fontan conduit [90]. Therefore, a thorough prior evaluation with multimodal imaging, especially 3D transesophageal echocardiography and computed tomography imaging, is crucial. Additionally, the use of 3D anatomical models can be useful for the planning and anatomical comprehension of these procedures. TEER in Fontan patient using MitraClip (Abbott Vascular, US) has reported successful percutaneous interventions, however, long-term results are unknown [91,92].

#### 3.5.2. Residual Ventriculo-Pulmonary Communication Transcatheter Closure

There are doubts about the benefit of maintaining restrictive antegrade pulmonary blood flow through a stenotic pulmonary outflow tract after the bidirectional Glenn procedure [93]. However, when the Fontan procedure is completed, it is crucial to eliminate any additional blood flow from the ventricle to the pulmonary arteries to prevent volume overload of the heart, as its persistence has been associated with persistent pleural effusions and progressive ventricular failure [94]. Occasionally, a residual surgical defect may result in a persistent antegrade connection between the single ventricle and the pulmonary circulation. This can be easily identified by the presence of pulsatility in the pulmonary artery and confirmed through pulmonary angiography. Although generally small, these defects can compromise Fontan circulation, so their percutaneous closure is feasible and recommended using various occlusion devices [94].

#### 3.5.3. Thrombectomy in Acute Pulmonary Thromboembolic Disease

**Figure 6:**
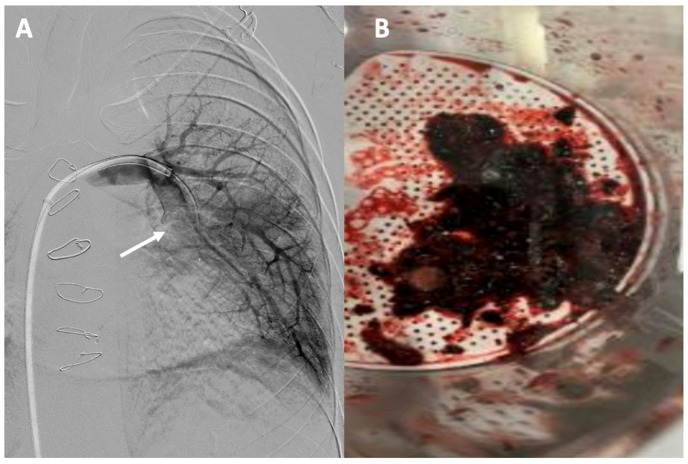
Acute pulmonary thromboembolism: (**A**) angiographic image of a thrombotic occlusion in the main branch of the left lower lobar artery (see arrow); (**B**) thrombus extracted after percutaneous aspiration thrombectomy.

Adult Fontan patients face a significant risk of thromboembolic complications [95]. One of the primary limitations lies in diagnosis as well as risk stratification. Conventional methods such as electrocardiography, echocardiography, and changes in troponin levels are less useful due to the absence of a right ventricle. The use of computed tomography angiography for the initial diagnosis of pulmonary embolism in patients with Fontan circulation is essential but often presents challenges [96]. Defects in contrast filling due to alterations in the venous phase are caused by the lack of connection between the right ventricle and the lung, as well as the typical asymmetry in pulmonary flows. Therefore, it is necessary to consider different strategies when acquiring images, such as using a dual contrast injection in both the upper and lower extremities and obtaining biphasic images to facilitate pulmonary opacification [97,98]. Various percutaneous options have been developed for the treatment of pulmonary embolism based on techniques such as mechanical thrombectomy, aspiration thrombectomy, and catheter-directed thrombolysis [99]. Although the indications for such therapies in this population are not clearly defined, there have been satisfactory cases of thromboaspiration in Fontan patients [100] (Figure 6).

#### 3.5.4. Aortic Coarctation Repair

**Figure 7:**
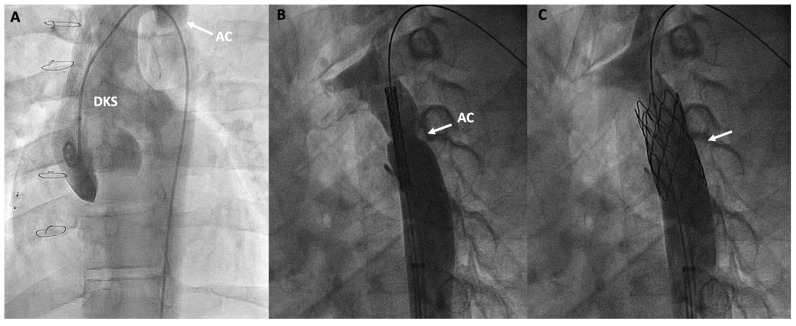
Double inlet left ventricle, initially palliated with a Damus-Kaye-Stansel (DKS) procedure and subsequently with a Fontan procedure, presenting an invasive gradient of 18 mmHg in the thoracic aorta. (**A**) Angiographic image of the neoaorta in a patient with DKS with an associated aortic coarctation (AC) marked by an arrow; (**B**) Placement of a stent at the level of the coarctation (arrow); (**C**) Percutaneous repair of the AC after stent deployment (arrow).

The presence of aortic coarctations or obstructions at the aortic arch can increase afterload and compromise the proper functioning of the Fontan circulation. Therefore, repair may be advised even with small gradients [19]. There is a subset of patients with Fontan circulation, such as those previously palliated with the Norwood procedure and the less common Damus-Kaye-Stansel surgery, who may experience early coarctation issues in the neo-aorta more frequently in the first few years of life [101]. However, exceptional cases of recoarctation can be observed in adulthood. Transcatheter treatment of aortic coarctation or aortic arch obstruction primarily involves balloon angioplasty, which, despite providing early improvement, has a high recurrence rate, or stent implantation, which has been shown to provide good long-term results [19] (Figure 7).

#### 3.5.5. Management of Intrapulmonary Shunt

Intrapulmonary shunting due to PAVM can be a serious complication that causes cyanosis and an increased risk of paradoxical emboli and pulmonary hemorrhage [54]. It has been observed that hepatic flow to the pulmonary arteries is crucial in preventing these PAVMs. There is a hypothesis concerning an unidentified “hepatic actor” which regulates pulmonary angiogenesis, and the lack of hepatic flow to the pulmonary artery leads to the development of PAVMs [102].

Reducing the risk of pulmonary arteriovenous fistulas has been associated with the implementation of bidirectional cavopulmonary anastomosis and promoting the early completion of Fontan surgery to ensure hepatic flow in patients with a prior bidirectional Glenn procedure [103]. Conceptually, once PAVM are detected after Fontan completion, an obstruction in the pathway from the inferior vena cava to the pulmonary artery should be ruled out and treated if present. However, there are two particular scenarios where arteriovenous fistulas can develop with high prevalence: (1) in patients with unbalanced pulmonary artery flow from the hepatic vein [54], and (2) in patients with left isomerism who have undergone a Kawashima procedure followed by Fontan surgery [104]. This last scenario is attributed to difficulties in equalizing hepatic flow distribution to both pulmonary arteries in cases where there is minimal hepatic flow compared to the larger systemic venous flow from the superior vena cava.

Therapeutic options vary based on the nature and distribution of the fistulas. For discrete arteriovenous fistulas, closure can be achieved using occluder devices, although PAVMs usually have a diffuse distribution that complicates this approach [103]. Various interventions have been proposed to redistribute hepatic flow more evenly to the pulmonary arteries, thereby increasing the “hepatic factor” and facilitating the regression of the pulmonary arteriovenous fistulas. One option is the creation of an endovascular peripheral arteriovenous fistula which would deliver “hepatic factor” to the pulmonary circulation through the bidirectional Glenn anastomosis [105]. More complex alternatives include establishing a transcatheter connection from the hepatic duct to the azygos vein, which has proven useful in reversing these malformations in patients with heterotaxy and an interrupted inferior vena cava [106].

#### 3.5.6. Percutaneous Fontan Completion

For years, several hybrid strategies have been developed with the aim of completing the Fontan circulation percutaneously thus reducing the need for surgical interventions. At the time of creating the bidirectional Glenn, a preparatory staging is performed for its subsequent transcatheter completion [107]. The surgical technique consists of creating a lateral tunnel with a single large fenestration that communicates with the common atrium, closing the communication with the superior vena cava with a patch, thus maintaining the physiology of the bidirectional Glenn. Subsequently, in a second stage, the Fontan could be completed percutaneously by perforating the patch using radiofrequency, stenting it, and closing the fenestration with an occlusion device. Several experiences have been described but their uses have not been generalized [108,109]. New prototypes are being developed using an extracardiac-type Fontan circuit pathway [110,111].

## 4. Gaps in Knowledge and Future Directions

As we look at the recent advances in managing Fontan circulation, several key areas of research and development hold promise. However, significant knowledge gaps remain.

### 4.1. Diagnostic Catheterization

One of the unresolved questions includes why pulmonary vascular disease is associated with long-term Fontans, and which patients might benefit from pulmonary vasodilator therapy. It has been observed that, unlike classic pulmonary hypertension, characterized by pulmonary artery medial hypertrophy, Fontan patients with PVD exhibit a distinct pattern of intimal hyperplasia and medial smooth muscle cell regression. It is hypothesized that the non-pulsatile, low-shear flow promotes increased apoptosis of vascular smooth muscle cells in the medial layer [40]. Furthermore, this phenomenon has been detected both in the intra-acinar pulmonary vessels and in the tissues of the main pulmonary artery [112]. The combined use of CPET and catheterization may be particularly useful for the early diagnosis of poor pulmonary vascular reserve [43]. However, early data suggest that intravascular imaging techniques such as optical coherence tomography (OCT) could serve as a valuable tool for studying vascular remodeling in Fontan patients. This technology may help identify diverse patterns of adverse pulmonary remodeling, which could have significant therapeutic implications [113]. Given the unconvincing outcomes of recent clinical trials with conventional pulmonary vasodilator drugs [114], which primarily target smooth muscle cells, it is hypothesized that novel drugs with antiproliferative properties may yield better results for this patient subset.

Another important area of uncertainty affects cases of segmental pulmonary hypertension. The combined use of cardiac MR and catheterization can provide valuable information for selected patients in calculating flow dependent on large and non-restrictive MAPCAs, and estimating resistances in each segment [37,38]. This can be particularly useful for those considering heart transplantation or pulmonary vasodilator therapy.

In contrast to systolic ventricular failure, which is typically identified through imaging findings, diastolic ventricular dysfunction has likely been underrecognized as a cause of heart failure, and in some cases may require advanced therapies. The mechanisms underlying diastolic function in single ventricles remain poorly understood, and the geometric variability between right and left ventricular morphology further complicates its understanding [115]. Recent studies have provided useful information on stress maneuvers, such as fluid challenge and especially exercise tests to detect early-stage diastolic dysfunction [44,48]. However, the analysis of classic pressure-volume loops, which still presents technical challenges, could provide valuable insights into ventricular function and may represent a promising area for future research [115].

The prognostic stratification of the impact of Fontan physiology on liver function through invasive hemodynamic studies remains very limited. Although central venous pressure is recognized as a relevant factor, HVPG does not appear to be useful in identifying patients with extensive FALD [24]. Other invasive hemodynamic parameters should be investigated to enhance the assessment and prediction of FALD outcomes, thereby aiding in the complex decision-making process regarding heart or heart-liver transplantation when necessary.

### 4.2. Interventional Catheterization

Most percutaneous interventions are currently focused on treating obstructions in the Fontan pathway or on embolizing different types of collaterals. However, innovative techniques are being developed to address less frequent but clinically challenging situations, such as the treatment of lymphatic disorders or redirecting hepatic venous flow in specific patients with diffuse PAVM [85,86,87,106]. Despite this progress, these therapies remain highly selective, and there are currently no clear indications. Perhaps, the most promising is the use of edge-to-edge valve repair therapy [91,92]. This technique still presents considerable challenges in terms of accessing the systemic atrium and the anatomical variability of the atrioventricular valve in these patients. Future studies in this area will be interesting to determine long-term outcomes and efficacy, potentially providing earlier indications that are currently limited by surgical risk. This could delay the need for heart transplantation in patients with systemic atrioventricular valve regurgitation.

For more than two decades, efforts have been aimed at minimizing the number of surgeries for these patients, and some centers have tried a percutaneous completion of the Fontan circuit. All of these techniques involve surgical preconditioning during the bidirectional Glenn operation. Both intracardiac and extracardiac transcatheter Fontan completions have been performed [108,109,110,111]. However, these experiences are limited to dedicated centers that report good results in selective populations. Although promising and very attractive, its generalization seems distant.

Lastly, initial designs of right heart assist devices, such as axial blood pumps or dual-lumen cannulas connected to extracorporeal pumps, have been proposed to improve hemodynamics in Fontan circulation, and have been tested in animal models [116,117]. In the past few years, a novel right heart assist device made of flexible materials has been developed, offering minimally invasive implantation and showing promising simulation results [118]. However, there is still no human experience, and issues such as the impact on blood pressure rise, hemolysis risk, and thrombus formation still need to be studied. This field represents a highly promising direction that could potentially enhance Fontan physiology. Further studies are needed to address the remaining challenges and ensure successful implementation in clinical practice.

## 5. Conclusions

Managing Fontan circulation remains a significant challenge due to the complexity of its physiology and the multiple associated complications. Despite the continuous development of interventional techniques, standardization is complex given the intrinsic variability of these patients, and clinical application must be individualized. The ongoing development of innovative percutaneous interventions is also crucial to address specific complications and improve long-term outcomes in patients with Fontan circulation. In the near future, diagnostic cardiac catheterization will play a crucial role in addressing unresolved challenges such as pulmonary vascular remodeling and diastolic ventricular dysfunction. Regarding interventional catheterization, in addition to the continued advancement of innovative techniques for treating systemic AV regurgitation and lymphatic disorders, our aspirations turn to percutaneous implantable collapsible axial blood pumps to address the primary limitation of Fontan physiology: the absence of a subpulmonary pump.

## Figures and Tables

**Table 1:** Diagnostic tools summary in cardiac catheterization for adults with Fontan circulation.

Diagnostic Tool	Description
**RHC**	Assessment of pressures, cardiac output, and vascular resistance to establish a hemodynamic profile at rest.
**Fluid challenge**	A test involving the rapid infusion of saline to evaluate the response of ventricular filling pressures and uncover potential hidden diastolic dysfunction.
**Passive leg lifting**	A maneuver that induces a moderate increase in cardiac output and can reveal potential hidden diastolic dysfunction.
**CPET combined with RHC**	It combines right heart catheterization with a cardiopulmonary exercise test using a supine cycling protocol to identify hemodynamic patterns such as hidden diastolic dysfunction, pulmonary vascular impairment, inability to increase cardiac output, and Fontan pathway obstruction.
**Angio MR combined with RHC**	Specific protocols combine angio MR and cardiac catheterization to determine flows dependent on large MAPCAs and accurately calculate segmental resistances.
**Angiographies**	Administration of iodinated contrast to anatomically evaluate the Fontan pathway and rule out potential obstructions, as well as SPVCs, fenestrations, and MAPCAs.
**Bubbles contrast**	Selective administration of agitated saline into the pulmonary branches, combined with echocardiography, to detect bubble passage into the cardiac chambers in the presence of PAVMs.

CMRI: Cardiac Magnetic Resonance Imaging, CPET: Cardiopulmonary Exercise Test, MAPCAs: Major Aortopulmonary Collateral Arteries, PAVMs: Pulmonary Arteriovenous Malformations, RHC: Right Heart Catheterization, SPVCs: Systemic-to-Pulmonary Venous Collaterals.

**Table 2:** Proposed abnormal cutoff points for hemodynamic assessment in adult Fontan patients.

At Rest	
Fontan pressure (central venous pressure)	>15 mmHg
PAWP	>12 mmHg
IVC-SVC gradient pressure	≥2 mmHg
Transpulmonary gradient pressure	≥7 mmHg
Cardiac index	<2.5 L/min/m^2^
Pulmonary vascular resistance index	>2 WU*m^2^
Transaortic arch gradient pressure	>10 mmHg
**At Exercise**	
Fontan pressure (central venous pressure)	>25 mmHg
PWAP	>20 mmHg
IVC-SVC gradient	≥5 mmHg
Slope mPAP/CO pulmonary	>3 mmHg/L/min
Slope PAWP/CO systemic	>2 mmHg/L/min

CI: Cardiac Index, CVP: Central Venous Pressure (Fontan Pressure), IVC-SVC: Inferior Vena Cava-Superior Vena Cava Gradient Pressure, mPAP/CO: Slope Mean Pulmonary Arterial Pressure/Cardiac Output Pulmonary, PAWP/CO: Slope Pulmonary Arterial Wedge Pressure/Cardiac Output Systemic, PVRi: Pulmonary Vascular Resistance Index, PWAP: Pulmonary Wedge Atrial Pressure.

**Table 3:** Interventional therapeutic options for adult Fontan circulation.

Percutaneous Interventions	Description
Fontan Pathway Obstructions	Balloon angioplasty, and especially stenting, are commonly used to treat obstructions in the Fontan pathway and pulmonary branch stenosis.
Fenestrations	Fenestration can be created by puncture, usually with subsequent stent implantation, or more commonly, closed percutaneously using transcatheter occlusion devices.
Collaterals	MAPCAs and SPVCs can be treated by embolization (using coils, microspheres, or liquid embolic systems), or occluded with vascular plug devices.
Lymphatic Circulation	Selective lymphatic embolization and percutaneous thoracic duct decompression can be used to manage lymphatic disorders.
AV regurgitation	TEER therapy using MitraClip^®^ device can be useful in treating selected cases of atrioventricular valve insufficiency.
Ventriculo-pulmonary Communication	Residual antegrade connections between the ventricle and pulmonary circulation can be closed percutaneously using occlusion devices.
Acute Pulmonary Thromboembolic Disease	Mechanical thrombectomy and aspiration thrombectomy may be useful for treating pulmonary embolism in selected cases of Fontan patients.
Aortic Coarctation	Balloon angioplasty and, more frequently, stent implantation can be used to repair aortic arch and aortic coarctations, which can compromise Fontan circulation.
Intrapulmonary Shunt	Different techniques for redirecting hepatic flow to the pulmonary circulation could be useful in selected patients with severe intrapulmonary shunt in the context of diffuse PAVMs.
Percutaneous Fontan Completion	Hybrid strategies for completing the Fontan circulation percutaneously, primarily intracardiac, have been described with the goal of reducing the number of surgeries.

AV: Atrioventricular, MAPCAs: Major Aortopulmonary Collateral Arteries, PAVMs: Pulmonary Arteriovenous Malformations, SPVCs: Systemic-to-Pulmonary Venous Collaterals, TEER: Transcatheter Edge-to-Edge Repair.

## Abbreviations

CI	Cardiac index
CO	Cardiac output
CPET	Cardiopulmonary exercise test
FALD	Fontan-associated liver disease
HVPG	Hepatic venous pressure gradient
IH-DCMRL	Intrahepatic dynamic contrast magnetic resonance lymphangiography
IN-DCMRL	Intranodal dynamic contrast magnetic resonance lymphangiography
IVC-SVC gradient	Inferior vena cava—superior vena cava gradient
MAPCAs	Major aortopulmonary collateral arteries
MR	Magnetic resonance
PAVM	Pulmonary arteriovenous malformations
PAWP	Pulmonary artery wedge pressure
PVR	Pulmonary vascular resistance
PVRI	Indexed pulmonary vascular resistance
SPVCs	Systemic-to-pulmonary venous collaterals
TEER	Transcatheter edge-to-edge repair
TPG	Transpulmonary gradient
VO_2_	Oxygen consumption
